# Supplementary material for: Reducing respiratory syncytial virus (RSV) hospitalization in a lower-income country by vaccinating mothers-to-be and their households
Source: eLife. 2020 Mar 27;9:e47003. doi: 10.7554/eLife.47003 (PMC7556875; doi:10.7554/eLife.47003)
Supplement: Transparent reporting form [file elife-47003-transrepform.docx]

***eLife’s* transparent reporting form**

We encourage authors to provide detailed information *within their submission* to facilitate the interpretation and replication of experiments. Authors can upload supporting documentation to indicate the use of appropriate reporting guidelines for health-related research (see [EQUATOR Network](http://www.equator-network.org/%20)), life science research (see the [BioSharing Information Resource](https://biosharing.org/)), or the [ARRIVE guidelines](http://www.plosbiology.org/article/info:doi/10.1371/journal.pbio.1000412) for reporting work involving animal research. Where applicable, authors should refer to any relevant reporting standards documents in this form.

If you have any questions, please consult our Journal Policies and/or contact us: [editorial@elifesciences.org](mailto:editorial@elifesciences.org).

**Sample-size estimation**

- You should state whether an appropriate sample size was computed when the study was being designed
- You should state the statistical method of sample size computation and any required assumptions
- If no explicit power analysis was used, you should describe how you decided what sample (replicate) size (number) to use

Please outline where this information can be found within the submission (e.g., sections or figure legends), or explain why this information doesn’t apply to your submission:

This paper describes how demographic, known epidemiological and RSV incidence data can be combined to make model-based inference of unknown transmission and epidemiological parameters. And then use this inference to forecast different RSV vaccination scenarios.

Because it is a model-based approach we didn’t need to pre-plan the sample size required.

**Replicates**

- You should report how often each experiment was performed
- You should include a definition of biological versus technical replication
- The data obtained should be provided and sufficient information should be provided to indicate the number of independent biological and/or technical replicates
- If you encountered any outliers, you should describe how these were handled
- Criteria for exclusion/inclusion of data should be clearly stated
- High-throughput sequence data should be uploaded before submission, with a private link for reviewers provided (these are available from both GEO and ArrayExpress)

Please outline where this information can be found within the submission (e.g., sections or figure legends), or explain why this information doesn’t apply to your submission:

As above, this is a model-based inference and forecasting paper, hence biological experiments were not performed.

**Statistical reporting**

- Statistical analysis methods should be described and justified
- Raw data should be presented in figures whenever informative to do so (typically when N per group is less than 10)
- For each experiment, you should identify the statistical tests used, exact values of N, definitions of center, methods of multiple test correction, and dispersion and precision measures (e.g., mean, median, SD, SEM, confidence intervals; and, for the major substantive results, a measure of effect size (e.g., Pearson's r, Cohen's d)
- Report exact p-values wherever possible alongside the summary statistics and 95% confidence intervals. These should be reported for all key questions and not only when the p-value is less than 0.05.

Please outline where this information can be found within the submission (e.g., sections or figure legends), or explain why this information doesn’t apply to your submission:

We used a maximum likelihood approach to inferring the six missing parameters of the RSV transmission model (parameters described in l. 527-533 main document). We used a Poisson link function between the continuous variables of the RSV transmission model and the count data from KCH hospitalizations, a standard approach for modelling count data (equation (8) main document).

We treat the variation in the seasonality of RSV in Kilifi as being due to unobserved latent random effects (appendix 1 of main document), this approach is completely standard for mixed-effects regression (e.g. for generalized mixed-effect linear models), although more unusual for transmission modelling. However, we achieved maximum likelihood estimates for models with unobserved latent variables using the EM algorithm, which is standard method for inference problems with unobserved variables (see **Parameter Inference** subsection in main document, and **Parameter inference for the household- and age- model** in supporting information for details of EM algorithm implementation). 95% CIs for each parameter were constructed by considering the likelihood profile region around the maximum point where the decrease in log-likelihood by univariate variation of the parameter was smaller than **c/2** where c is defined by the distribution function of a chi-squared random variable with one degree of freedom, Prob($\chi_{1}^{2}<c)=0.95$, again this is a standard method for constructing 95% Cis when a log-likelihood profile is available (see table 3 supporting information and l. 216 main document).

As mentioned above, the RSV transmission model in this paper uses continuous variables. We interpret the model predicted continuous cumulative incidence of hospitalization (see **Hospitalization rates** subsection in main document, and table 2 supporting information for age-dependent hospitalization rates per infection episode) as the expected number of a Poisson distributed number of hospitalizations. This approach allowed likelihood-based inference, and additionally, the Poisson number of hospitalizations assumption allowed us to construct standard prediction intervals for weekly numbers of hospitalizations. In Fig. 2 the prediction, and prediction intervals, of the model after inference is shown as visual comparison to the true data. In Fig. 5 the prediction, and prediction intervals, of the forecasts are shown.

When forecasting the effect of vaccination at each level of coverage we presented the median percentage reduction in either hospitalizations or infecteds compared to a baseline of no-intervention over 500 realizations of the future seasonality random effects (Fig. 4 and Fig 6.). To minimize monte-carlo variance we used the same 500 realizations for each level of coverage; that is that each forecast is a comparison to a baseline with **identical** seasonal trends.

(For large datasets, or papers with a very large number of statistical tests, you may upload a single table file with tests, Ns, etc., with reference to sections in the manuscript.)

**Group allocation**

- Indicate how samples were allocated into experimental groups (in the case of clinical studies, please specify allocation to treatment method); if randomization was used, please also state if restricted randomization was applied
- Indicate if masking was used during group allocation, data collection and/or data analysis

Please outline where this information can be found within the submission (e.g., sections or figure legends), or explain why this information doesn’t apply to your submission:

As above, this is a model-based inference and forecasting paper, hence biological experiments were not performed.

**Additional data files (“source data”)**

- We encourage you to upload relevant additional data files, such as numerical data that are represented as a graph in a figure, or as a summary table
- Where provided, these should be in the most useful format, and they can be uploaded as “Source data” files linked to a main figure or table
- Include model definition files including the full list of parameters used
- Include code used for data analysis (e.g., R, MatLab)
- Avoid stating that data files are “available upon request”

Please indicate the figures or tables for which source data files have been provided:

We have included the MATLAB® script which generated each of figures 2-6 in the main document along with the necessary data files and functions for generating plots. This has been submitted as “Plots.zip”.

For full model code, please see the linked Github repository https://github.com/SamuelBrand1/RSVHouseholdModel.git
